# Lipid removal in deuterium metabolic imaging (DMI) using spatial prior knowledge

**DOI:** 10.5194/mr-5-21-2024

**Published:** 2024-04-09

**Authors:** Robin A. de Graaf, Yanning Liu, Zachary A. Corbin, Henk M. De Feyter

**Affiliations:** 1 Department of Radiology and Biomedical Imaging, Magnetic Resonance Research Center, Yale University School of Medicine, New Haven, Connecticut, USA; 2 Biomedical Engineering, Magnetic Resonance Research Center, Yale University School of Medicine, New Haven, Connecticut, USA; 3 Neurology, Magnetic Resonance Research Center, Yale University School of Medicine, New Haven, Connecticut, USA

## Abstract

Deuterium metabolic imaging (DMI) is a novel method to generate spatial maps depicting dynamic metabolism of deuterated substrates, such as [6,6^′^-^2^H_2_]-glucose, and their metabolic products, like ^2^H-lactate. While DMI acquisition methods are simple and robust, DMI processing still requires expert user interaction, e.g., in the removal of extracranial natural abundance ^2^H lipid signals that interfere with metabolism-linked ^2^H-lactate formation. Here we pursue the use of MRI-based spatial prior knowledge on brain and non-brain/skull locations to provide robust and objective lipid removal. Magnetic field heterogeneity was accounted for using DMI-derived surrogate B_0_ and B_1_ maps, as well as through subdivision of the skull region into smaller compartments. Adequate lipid removal with an average suppression of 90.5 ± 11.4 % is achieved on human brain in vivo without perturbation of the metabolic profile in brain voxels, thereby allowing for the generation of distinct and reliable metabolic maps for patients with brain tumors.

## 1 Introduction

Deuterium metabolic imaging (DMI) is a recent method to map the spatial distribution of ^2^H-enriched substrates and their metabolic products in health and disease (De Feyter et al., 2018; Kaggie et al., 2022; Adamson et al., 2023). The most commonly used substrate, [6,6^′^-^2^H_2_]-glucose, has shown promise to offer unique metabolic insights into brain tumors (De Feyter et al., 2018; Adamson et al., 2023), stroke (Straathof et al., 2021), brown adipose tissue (Riis-Vestergaard et al., 2020), heart (Wang et al., 2021), preeclampsia (Markovic et al., 2021), and a range of tumors outside the brain (Kreis et al., 2020; Veltien et al., 2021). Other ^2^H-enriched substrates, such as ^2^H_9_-choline and [2,3-^2^H_2_]-fumarate, can provide insights into tumor proliferation (Veltien et al., 2021; Ip et al., 2023) and cell death (Hesse et al., 2021), respectively. DMI sets itself apart from other metabolic imaging modalities such as ^1^H, ^13^C, hyperpolarized ^13^C, and ^31^P MRSI (magnetic resonance spectroscopy imaging) through its simple and robust acquisition methods. The low natural abundance of deuterium eliminates the need for water and lipid suppression, whereas the sparsity of ^2^H MR spectra reduces the magnetic field homogeneity requirements, thereby enabling the acquisition of 3D DMI across the entire human head with high-quality spectra at all locations (De Feyter et al., 2018; Ruhm et al., 2021; Liu et al., 2022; Seres Roig et al., 2023).

Processing of DMI is generally straightforward, with high-quality quantification achieved using least-squares curve fitting with a limited number of Lorentzian lines (De Feyter et al., 2018). DMI of [6,6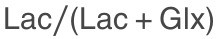 provides high-contrast images of pathological metabolism as described for brain and other tumors (De Feyter et al., 2018), as well as stroke (Straathof et al., 2021). With sufficient sensitivity, as seen near radiofrequency (RF) coil receive elements, natural abundance ^2^H lipid signals originating from the skull will produce a detectable MR signal. While these small lipid signals do not cause the widespread contamination throughout the brain as seen for ^1^H MRSI (Tkáč et al., 2021), they can lead to artifactual intensity in Lac and Lac-Glx ratio maps as the Lac and lipid signals share near-identical chemical shifts. Lipid suppression in human brain MRSI studies can be achieved through pulse sequence modifications (inner volume selection, outer volume suppression, longer echo times), additional hardware (higher-order gradient coils, crusher coils), or post-processing methods. While pulse sequence modifications and hardware solutions can give excellent lipid suppression (Tkáč et al., 2021), the requirements for DMI are generally modest, making post-processing methods a logical choice while still retaining the simple DMI acquisition method. Many post-processing methods utilize MRI-based prior knowledge on the lipid spatial location to achieve lipid removal and include dual-density reconstruction (Hu et al., 1994; Metzger et al., 1999), data extrapolation (Haupt et al., 1996), L2 regularization (Bilgic et al., 2014), and spectral localization by imaging (SLIM; Hu et al., 1988) and its variants (Liang and Lauterbur, 1991; Von Kienlin and Mejia, 1991; Bashir and Yablonskiy, 2006; Khalidov et al., 2007; Zhang et al., 2012; Passeri et al., 2014; Adany et al., 2016, 2021).

Here we propose to use the SLIM algorithm (Hu et al., 1988) because it can (1) be applied to standard 3D phase-encoded DMI without data acquisition modifications, (2) remove extracranial lipids without perturbing the brain metabolic profile, and (3) be extended to provide regional brain signals from anatomy-matched compartments. The processing pipeline, including MRI brain/skull segmentation, generation of DMI-based surrogate B_0_ and B_1_ maps, and SLIM-based regional signal removal, can be fully automated and provides a robust and objective tool to accelerate the inclusion of DMI in a clinical MR workflow.

## 2 Methods

### 2.1 Methods

SLIM belongs to a class of post-processing methods that constrains the MRSI reconstruction with spatial prior knowledge derived from anatomical MRIs. The MRSI acquisition is formulated as a linear model whereby the acquired *k*-space data **P** represents the linear sum of region of interest (ROI)-specific signals **C** weighted by a gradient and position-dependent factor **G** according to
1
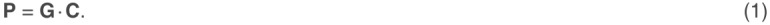


**P** is the measured *N*_enc_ × *N*_time_ *k*-space data matrix, with *N*_enc_ representing the total number of phase-encoding gradient combinations (i.e., number of *k*-space encodings) and *N*_time_ representing the number of complex, spectroscopic time domain points. **C** is an *N*_ROI_ × *N*_time_ matrix containing the time-domain signals from each of the *N*_ROI_ compartments, the sum of which comprises the entire object under investigation (e.g., human head, including brain and skull areas). The shape of the ROIs can be arbitrary with *N*_ROI_≤*N*_enc_. **G** is an *N*_enc_ × *N*_ROI_ encoding matrix describing the amount of signal dephasing across a given compartment *k*, during phase-encoding step *m*, according to
2
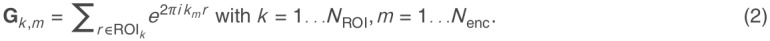

*k*_*m*_ represents the time integral of phase-encoding gradient *m*. The SLIM algorithm calculates the ROI-specific signal **C** from the measured data **P** according to
3
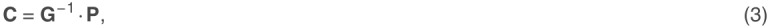

whereby the inverse of **G** can be obtained through singular value decomposition (SVD). If the object under investigation can be decomposed into **C**_ROI_ *homogeneous* compartments, without any requirements on homogeneity *between* compartments, then the SLIM algorithm produces **C**_ROI_ signals from the ROIs without any contamination from other compartments.

**Figure 1 Ch1.F1:**
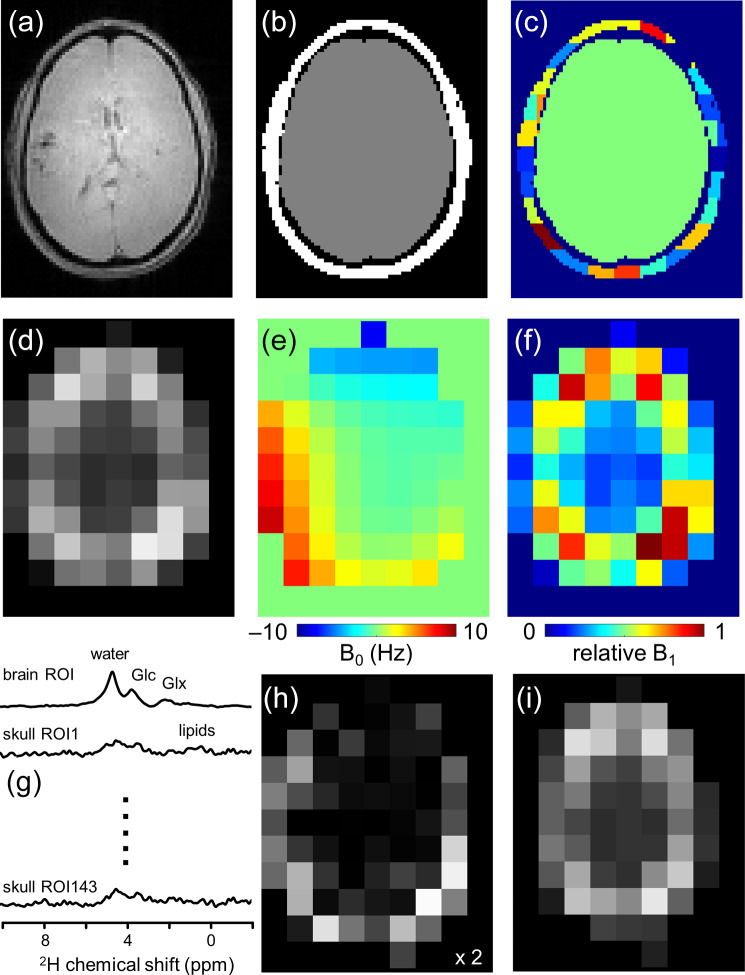
**Figure 1**Lipid removal workflow. **(a)** Anatomical MRI and **(b)** brain and non-brain/skull ROIs. **(c)** To accommodate heterogeneity in the skull ROI, it is subdivided into 125–175 ROIs of 3.5–8.5 mL. **(d)** ^2^H-water map obtained through numerical integration of each pixel in a 9 × 13 × 11 DMI dataset. **(e)** ^2^H-water line shift map and **(f)** relative ^2^H-water intensity map. **(g)** Compartment-specific signals from one brain ROI and 143 skull ROIs are the primary output of the SLIM algorithm. **(h)** DMI reconstructed from the 143 skull ROI signals, which can be subtracted from **(d)** the original DMI to yield **(i)** a skull-free (and lipid-free) DMI dataset.

Figure [Fig Ch1.F1] summarizes how the outlined algorithm can be utilized to achieve lipid removal in DMI. Based on anatomical MRIs (Fig. [Fig Ch1.F1]a), the human head can be segmented into *N*_brain_ ROIs and *N*_skull_ ROIs (Fig. [Fig Ch1.F1]b and c), after which the gradient encoding matrix **G** can be calculated for each ROI. With the measured DMI data (Fig. [Fig Ch1.F1]d) as input **P**, the ROI-specific signals **C** can be calculated via Eq. ([Disp-formula Ch1.E3]) (Fig. [Fig Ch1.F1]g). The *N*_skull_ signals can be used to reconstruct, via Eq. ([Disp-formula Ch1.E1]), a skull MRSI dataset (Fig. [Fig Ch1.F1]h), which can be subtracted from the original MRSI to provide a skull-free, lipid-suppressed MRSI dataset (Fig. [Fig Ch1.F1]i).

When all ROIs are homogeneous, the suppression of skull-based signals is expected to be perfect. Unfortunately, the requirement for homogeneous compartments is rarely encountered experimentally due to variations in metabolic composition and B_0_ and B_1_ magnetic fields across the sample. This heterogeneity is a violation of the linear model (Eq. [Disp-formula Ch1.E1]) and will lead to contamination (or “bleeding”) between ROIs (Liang and Lauterbur, 1993; Von Kienlin and Mejia, 1991) and thus an incomplete removal of skull-based lipids. Several strategies have been developed to address the issue of signal heterogeneity. Multiple methods incorporate prior knowledge on magnetic field heterogeneity from B_0_ and/or B_1_ maps into the SLIM processing pipeline (Bashir and Yablonskiy, 2006; Khalidov et al., 2007; Passeri et al., 2014; Adany et al., 2016). Other methods seek to optimize the *k*-space encoding scheme to minimize bleeding between ROIs (Von Kienlin and Mejia, 1991; Zhang et al., 2012), whereas another strategy rests on subdividing larger ROIs to decrease the heterogeneity across any one ROI (Adany et al., 2021; Dong and Hwang, 2006).

Here we employ two strategies, namely, (1) the subdivision of the non-brain ROI into smaller more homogeneous compartments and (2) the incorporation of prior knowledge on known B_0_ and B_1_ magnetic field heterogeneity. Following the segmentation of brain and non-brain ROIs, the latter is subdivided further (Fig. [Fig Ch1.F1]c) by multiplying the ROI with an equidistant 3D grid. A minimum ROI volume is enforced by combining adjacent ROIs for which at least one falls below a minimum threshold. The effect of the ROI size and number of ROIs on the localization performance can be quantitatively evaluated through calculation of the spatial response function (SRF; Von Kienlin and Mejia, 1991) according to
4
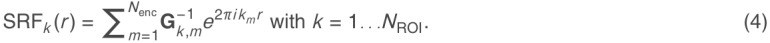


The SRF is a complex function that depicts the spatial extent of each ROI given a set of *k*-space encodings, whereby the net SRF contribution of a given ROI is zero across all other ROIs.

The incorporation of B_0_ and B_1_ magnetic field heterogeneity is achieved through a modification of the encoding matrix (Eq. [Disp-formula Ch1.E2]) according to
5
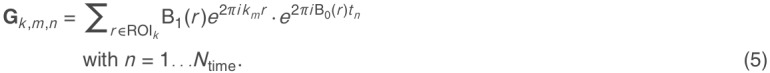


Note that 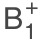 and receive 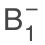 magnetic fields. While high-resolution B_0_ and B_1_ maps can be obtained with routine MR methods for ^1^H MRSI, the low-sensitivity DMI data prevent a comparable implementation. The calculation of surrogate B_0_ and B_1_ maps (Fig. [Fig Ch1.F1]e and f) from the measured DMI data will be described next.

### 2.2 Simulations

To evaluate the effectiveness of extracranial lipid suppression and intracranial metabolite retention, simulations were performed on brain and non-brain ROIs segmented from *T*_2_-weighted MRIs of five human subjects. To limit the overall calculation times, all simulations were performed on a single 2D slice. The brain ROIs were manually segmented into gray matter (GM), white matter (WM), and cerebrospinal fluid (CSF). A pathological ROI (e.g., tumor, stroke) with a random size (72 %–148 % of a nominal MRSI voxel), shape, and position was placed inside the brain ROI. With 10 random pathology ROI variations per subject, a total of 50 datasets were created for simulation.

Heterogeneity in the B_0_ and B_1_ magnetic fields can significantly affect the reconstruction performance, and simulations were extended with typical distributions found across the human head in vivo. As ^2^H-based B_0_ and B_1_ maps are not readily available, DMI-derived B_0_ and B_1_ maps were determined on five subjects by measuring the ^2^H water line shift and ^2^H water intensity in each pixel across a 2D slice, respectively. The maps were parameterized with a fourth-order 2D polynomial fit to set the B_0_ and B_1_ distribution average and range. Each of the 50 simulated datasets was constructed with unique B_0_ and B_1_ distributions, calculated from randomly selected polynomial coefficients characterizing the in vivo ranges. To further accommodate B_0_ and B_1_ heterogeneity, the skull ROI was divided into multiple smaller ROIs. The division was initiated by multiplying the skull ROI by a grid of uniform voxels (e.g., 20 mm × 20 mm). The resulting skull ROIs were combined with the nearest-neighbor ROI when the ROI volume was below a minimum threshold volume (e.g., 40 % of a nominal volume). By adjusting the grid size, the number of skull ROIs was varied from 1 to 50 to determine the optimal setting.

Using knowledge on the spatial ROIs, B_0_ and B_1_ distribution, and MRSI *k*-space encoding, DMI datasets were calculated as a 9 × 13 spatial matrix and 512 complex points acquired over a 1.0 kHz spectral width using a single, well-resolved resonance line per compartment in addition to a water signal present in all compartments. The linewidths were chosen based on previously reported in vivo values (De Feyter et al., 2018, *T*_2_ = 30 ms). Unless specified otherwise, the amplitude per unit volume was identical for all signals. Optional Gaussian noise could be added to the entire 9 × 13 × 512 time domain DMI dataset. SLIM processing (i.e., Eq. [Disp-formula Ch1.E3]) resulted in ROI-specific signals **C**, from which the non-brain ROI signals were selected to calculate a non-brain DMI dataset. Subtraction of the non-brain DMI dataset from the experimentally measured DMI produced a lipid-free, brain-only DMI dataset. All data analysis is based on numerical integration of the well-separated spectral MR signals representing water, lipids, and metabolites.

### 2.3 Human studies in vivo

All human studies were approved by the Yale University Institutional Review Board. All scans were performed on a 4 T Magnex magnet (Magnex Scientific Ltd.) interfaced to a Bruker Avance III HD spectrometer running on ParaVision 6 (Bruker Instruments). The system was equipped with 67 cm diameter Magnex gradients capable of switching 30 mT m^−1^ in 1.1 ms. RF transmission and reception were conducted with a 28.5 cm diameter transverse electromagnetic (TEM) volume coil tuned to the proton frequency (170.5 MHz) for MRI and shimming. Deuterium RF reception at 26.2 MHz was achieved with a four-coil phased array that was driven as a single RF coil during RF transmission. The four 8 cm × 10 cm rectangular ^2^H array elements were positioned equidistantly on an 18 cm × 25 cm elliptical former that was positioned within the ^1^H TEM coil.

DMI was acquired with a pulse-acquire method, extended with 3D phase-encoding according to a spherical 
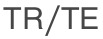
 
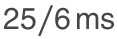
).

Processing of the in vivo DMI datasets is similar to that described for simulated data with a few noticeable differences. Firstly, whereas all in vivo DMI data are acquired as a 9 
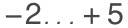
] Hz range) to the water linewidth. All spectral fitting was performed in DMIWizard, a graphical user interface programmed in MATLAB (The MathWorks, Natick, MA, USA) and freely available to the MR community (de Graaf, 2023). Finally, in the evaluation of metabolite retention, only pure-brain pixels are considered. For lipid suppression, partial skull pixels are also considered even though they can lead to an underestimation of the real suppression.

**Figure 2 Ch1.F2:**
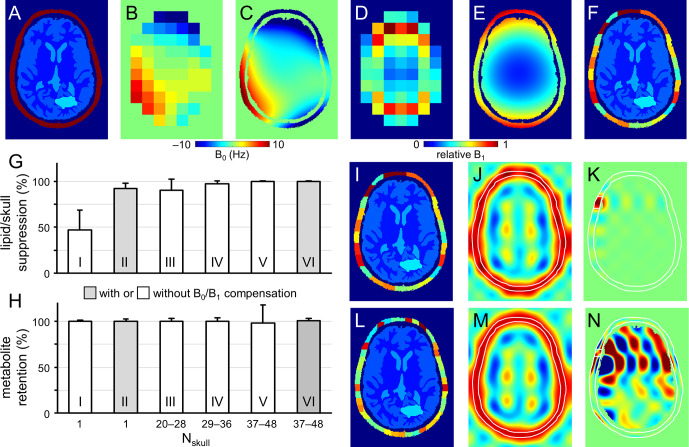
**Figure 2**Performance of SLIM-based lipid signal removal on phantoms in silico. **(a)** Brain and skull tissue constellation for 1 out of 50 permutations. **(b)** Water shift and **(d)** intensity maps as extracted from DMI data, providing **(c)** surrogate B_0_ and **(e)** B_1_ maps following low-order polynomial fitting. **(f)** ROI map with the skull ROI subdivided into 23 smaller compartments (i.e., *N*_skull_ = 23) to accommodate signal heterogeneity. **(g)** Lipid suppression and **(h)** metabolite retention under different scenarios including *N*_skull_ = 1 without (column I) and with (column II) B_0_ and B_1_ compensation, *N*_skull_ = 20–28 (column III), 29–36 (column IV), 37–48 (columns V and VI) without (columns III–V) and with B_0_ and B_1_ compensation (column VI). Results for the separate brain ROIs (GM, WM, CSF, and pathology) are summarized in Fig. S2 in the Supplement and essentially mirror the whole brain results shown in **(h)**. **(i, l)** Head constellations with the skull ROI subdivided into **(i)** 23 and **(l)** 44 smaller compartments. (**j**, **k**, **m**, and **n**) Spatial response function (SRF) of **(j, m)** the summed skull ROIs and **(k, n)** a single skull ROI for *N*_skull_ = 23 **(j, k)** and *N*_skull_ = 44 **(m, n)**. All SRFs have the same vertical scale, spanning −1 to +1. While the integrated *phase-sensitive* SRF intensity of a single skull ROI is zero across the brain ROI for any *N*_skull_, the integrated *absolute-valued* SRF intensity will be much larger for *N*_skull_ = 44 **(n)** compared to *N*_skull_ = 23 **(k)**. Figure S3 gives a summary of the absolute-valued SRF across the brain ROI for *N*_skull_ ranging from 1 to 50.

## 3 Results

Figure [Fig Ch1.F2]a shows the brain and non-brain ROIs used for one of the simulations, together with DMI-derived B_0_ (Fig. [Fig Ch1.F2]b) and B_1_ (Fig. [Fig Ch1.F2]d) maps, as well as surrogate B_0_ (Fig. [Fig Ch1.F2]c) and B_1_ (Fig. [Fig Ch1.F2]e) obtained by fitting the low-resolution maps in Fig. [Fig Ch1.F2]b and d with third-order 2D polynomials. The B_0_ magnetic field varied between −10 and +10 Hz over the areas analyzed, whereby the relative B_1_ amplitude varied from 0.15 in the center of the brain to 1.00 near the periphery.

Figure [Fig Ch1.F2]f shows the subdivision of the non-brain ROI into 23 smaller ROIs, whereby separate simulations investigated the effect of the number of non-brain ROIs. Figure S1 in the Supplement show five additional datasets used for simulation (out of a total of 50 datasets). All simulations achieved 100 % skull-based signal removal and 100 % brain signal retention in the absence of B_0_ and/or B_1_ heterogeneity. Even in the presence of B_0_ and B_1_ heterogeneity, perfect results were obtained provided that the *exact* B_0_ and B_1_ spatial distributions were incorporated according to Eq. ([Disp-formula Ch1.E5]). However, in the presence of B_0_ and/or B_1_ heterogeneity, skull-based signal suppression was only 47 ± 21 % when the skull ROI was not subdivided further (Fig. [Fig Ch1.F2]g, column I). When the reconstruction was supplemented with DMI-derived surrogate B_0_ and B_1_ maps, the skull-based signal suppression improved to 92 ± 6 % (Fig. [Fig Ch1.F2]g, column II). It was observed that the combined effect of incorporating surrogate B_0_ and B_1_ maps (6.6× reduction in residual lipids) was larger than that of the separate B_0_ (2.9× reduction) or B_1_ (1.1× reduction) maps. B_0_ compensation becomes less important, and the overall lipid suppression improves, as the spectroscopic linewidths become broader. Figure [Fig Ch1.F2]g and h (columns III to VI) explores the effect of subdividing the skull into *N*_skull_ ROIs, with *N*_skull_ ranging from 20–28 (Fig. [Fig Ch1.F2]g and h, column III), 29–36 (Fig. [Fig Ch1.F2]g and h, column IV), and 37–48 (Fig. [Fig Ch1.F2]g and h, columns V and VI). Without compensation for B_0_/B_1_ heterogeneity, the skull suppression increases with increasing *N*_skull_ as the smaller ROIs exhibit less heterogeneity across their volume (lipid suppression equals 90 ± 12, 97 ± 3, and 99 ± 1 % for Fig. [Fig Ch1.F2]g and h, column III–V). However, for higher *N*_skull_ the variability in metabolite retention increases from 3 % (Fig. [Fig Ch1.F2]g and h, column III) to 4 % (Fig. [Fig Ch1.F2]g and h, column IV) to 20 % (Fig. [Fig Ch1.F2]g and h, column V). Figure S2 in the Supplement summarizes metabolite brain retentions across separate brain ROIs (GM, WM, CSF, and pathology) and essentially mirror the trends seen across the entire brain (Fig. [Fig Ch1.F2]h). This effect can be understood by considering the SRF (Eq. [Disp-formula Ch1.E4]) as demonstrated for *N*_skull_ = 23 (Fig. [Fig Ch1.F2]i–k) and *N*_skull_ = 44 (Fig. [Fig Ch1.F2]l–n). In the case of *N*_skull_ = 23, both the overall skull SRF (Fig. [Fig Ch1.F2]j), being the phase-sensitive sum of all *N*_skull_ ROIs, and an exemplary single skull ROI (Fig. [Fig Ch1.F2]k) are well-behaved with the bulk SRF intensity within the intended skull ROI locations. Small contributions inside the brain have a zero net integral due to phase cancelation. In the case of *N*_skull_ = 44, the overall skull SRF (Fig. [Fig Ch1.F2]m) looks like that for *N*_skull_ = 23 (Fig. [Fig Ch1.F2]j). However, the single skull ROI (Fig. [Fig Ch1.F2]n) has the bulk SRF intensity inside the brain. Even though the integrated SRF intensity across the brain is zero, the strong reliance on phase-sensitive signal cancellation can lead to alteration of the brain metabolite signal distribution in the presence of compartmental heterogeneity, such as simulated in Fig. [Fig Ch1.F2]h (column V). Figure S3 in the Supplement summarizes the absolute-valued SRF brain contribution for every skull ROI when *N*_skull_ varies from 1 to 50. The SRF contribution across the brain ROI for any skull ROI is small and well-behaved for *N*_skull_ less than circa 35. However, when *N*_skull_ > 35, the SRF contribution sharply rises, resulting in localization that is highly dependent on phase cancelation – a condition that is violated in the presence of heterogeneity. When the B_0_/B_1_ heterogeneity is compensated with surrogate B_0_/B_1_ maps (Fig. [Fig Ch1.F2]h, column VI), the metabolite retention variability is greatly reduced (from 20 %, Fig. [Fig Ch1.F2]h, column V, to 3 %, Fig. [Fig Ch1.F2]h, column VI) as the large SRF intensity within the brain is properly phase-canceled. Overall, the simulation results in Fig. [Fig Ch1.F2] indicate that the effects of B_0_/B_1_ heterogeneity can be reduced through (1) compensation with surrogate B_0_/B_1_ maps, (2) subdivision of the skull region into smaller ROIs, or both, and that the skull ROI subdivision needs to balance improvement in skull suppression with increased variability in metabolite retention. Since the optimal ROI subdivision in Fig. S3 is fairly broad, all 3D human DMI data were divided according to a grid of nominal DMI voxels without further optimization. It should be noted that the brain ROI was not divided into smaller, tissue-specific ROIs (GM, WM, CSF, pathology) in any of the simulations. Preliminary simulations have shown that metabolite retention in separate tissue-specific ROIs is only marginally different if the brain is considered as one compartment (*N*_brain_ = 1, 98.9 ± 3.7 % across all tissue ROIs) or as four compartments (*N*_brain_ = 4, 99.7 ± 1.5 %). An intuitive explanation for this observation is that even though signal leakage between brain compartments does occur, the final step in the algorithm is the reconstruction and subtraction of a skull-only DMI dataset, leaving the brain signals largely unperturbed. It should also be noted that compartmental differences found in vivo are much smaller than those used in simulations (i.e., either signal or no signal), leading to reduced leakage. As the focus of the current work is on lipid removal, the decision was made to consider the brain as one compartment.

**Figure 3 Ch1.F3:**
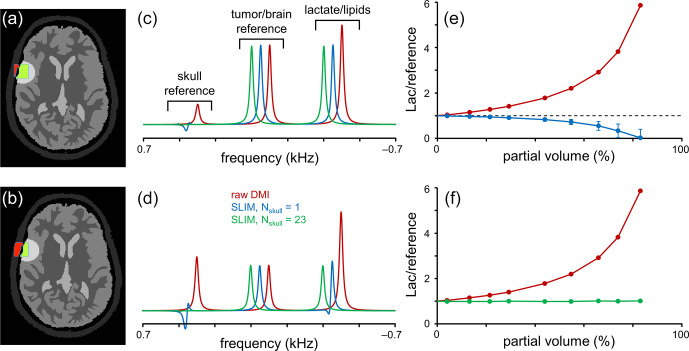
**Figure 3**Performance of SLIM-based lipid signal removal in the presence of partial volume averaging. **(a, b)** Brain, tumor, and skull ROI constellation used for all simulations. The amount of partial volume averaging is varied by shifting the ROIs relative to the MRSI grid, giving partial volumes of **(a)** 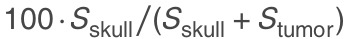 with *S*_skull_ (red in **a** and **b**) and *S*_tumor_ (green in **a** and **b**) representing the amount of signal from a reference signal specific for the skull and tumor ROIs, respectively. **(c, d)** MR spectra obtained from the MRSI voxel indicated in **(a, b)** with standard FFT processing (red) and SLIM-based processing without (blue) and with (green) skull ROI subdivision. For enhanced visualization, MR spectra are shifted by 50 Hz each. **(e, f)** Lactate-to-reference ratio as a function of partial volume for standard FFT processing (red) or SLIM processing without (blue) or with (green) skull subdivision. As the lipid removal without skull subdivision (blue) is compromised by B_0_ and B_1_ magnetic field homogeneity, the lactate-to-reference ratio changes with increasing partial volume. With skull subdivision (green), the lipid removal becomes less dependent on spatial homogeneity, leading to a stable lactate-to-reference ratio, independent of the partial volume. Error bars represent standard deviations calculated over 20 B_0_ and B_1_ magnetic field distributions. Note that the error bars for the red and green curves fall within the marker.

The results in Fig. [Fig Ch1.F2] were primarily focused on lipid/skull suppression and metabolite retention in voxel locations with minimal partial voluming. However, one of the most important clinical applications of the proposed algorithm is Lac retention in pathologies immediately adjacent to the skull where partial voluming can be significant. Figure [Fig Ch1.F3] summarizes Lac retention in the presence of partial voluming by using skull and brain-specific reference signals to monitor the amount of partial volume and the quality of lipid suppression.

The amount of partial voluming was adjusted by spatially shifting the ROI compartments relative to the fixed MRSI grid. Figure [Fig Ch1.F3]a and b show the ROI constellations to achieve ∼ 20 % (Fig. [Fig Ch1.F3]a) and ∼ 55 % (Fig. [Fig Ch1.F3]b) skull contribution to the indicated MRSI voxel. Figure [Fig Ch1.F3]c and d show MR spectra at the indicated MRSI voxel location extracted from a 2D MRSI dataset obtained with standard or SLIM processing. With standard FFT-based processing (red line), the spectra contain four signals corresponding to a skull reference signal, a brain/pathology reference signal (e.g., Glx), and a combined signal from Lac and lipids. As the reference signals have equal amplitudes per unit volume, the intensity of the skull and brain reference signals is indicative of the partial volume effect. After employing the SLIM algorithm with *N*_skull_ = 1 (blue line), the skull reference and lipid signals are largely removed. However, in agreement with Fig. [Fig Ch1.F2], the removal is incomplete in the presence of B_0_ and B_1_ heterogeneity, leading to an overestimation of the lipid signal and thus an underestimation of the Lac signal. Figure [Fig Ch1.F3]e shows the Lac-to-brain reference ratio as a function of the partial volume. With standard processing (red line), the ratio quickly increases as the large lipid contribution is included in the estimated Lac. With SLIM processing (*N*_skull_ = 1, blue line), the ratio stays close to one for small partial volumes but decreases as the lipid contribution is overestimated. When the SLIM algorithm is executed with skull subdivision (*N*_skull_ = 23, green line in Fig. [Fig Ch1.F3]c–f), the lipid removal becomes much less sensitive to B_0_ and B_1_ heterogeneity, leading to near-complete lipid removal and a correct Lac retention. The Lac-to-brain reference ratio stays close to one for all partial volumes (Fig. [Fig Ch1.F3]f). Note that a partial volume of 90 % only contains 10 % brain/pathology for which the ^2^H sensitivity would typically be too low to provide useful data.

**Figure 4 Ch1.F4:**
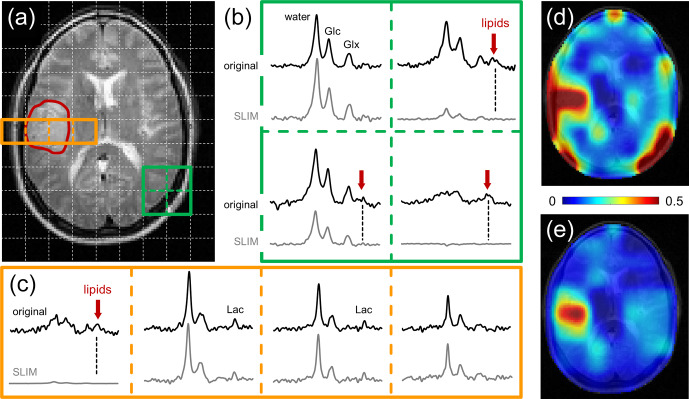
**Figure 4**Performance of SLIM-based lipid signal removal on human brain in vivo. **(a)** 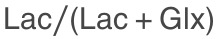 metabolic maps.

Figure [Fig Ch1.F4] shows the performance of SLIM-based skull removal on a patient with a glioblastoma tumor. Figure [Fig Ch1.F4]a shows a *T*_2_-weighted spin-echo MRI with the approximate tumor location outlined in red. The white dotted lines indicate the in-plane DMI grid (8 mL nominal resolution). Figure [Fig Ch1.F4]b shows a grid of ^2^H MR spectra extracted from the 3D DMI dataset at the location of the green 2 × 2 grid (Fig. [Fig Ch1.F4]a). The top spectra (black) represent the original, measured ^2^H MR spectra, whereas the bottom spectra (gray) are obtained following SLIM-based skull suppression. Signals from the pure skull voxel (lower right) are completely removed, whereas signals from the pure brain voxel (upper left) are fully retained. The two remaining voxel locations have both brain and skull contributions. The extracranial signals (primarily lipids, both also glucose and water) are suppressed, whereas the intracranial signals (glucose, glutamate, and water) are retained. Figure [Fig Ch1.F4]c shows a grid of ^2^H MR spectra extracted from the 3D DMI dataset at the location of the red 4 × 1 grid (Fig. [Fig Ch1.F4]a) through the tumor region. Only the skull voxel shows a pronounced lipid signal that is removed with SLIM processing. The remaining three spectra look visually identical with or without SLIM processing, with the increased lactate signal within the tumor region remaining visibly unperturbed. However, the removal of extracranial lipid signal has a significant effect on the resulting Lac/(Lac + Glx) metabolic maps (Fig. [Fig Ch1.F4]d and e). Without skull suppression, the lipid signals lead to several hot spots surrounding the brain (Fig. [Fig Ch1.F4]d), including an artifactual elevation of the lactate signals within the tumor. After SLIM-based skull removal, the lipid hot spots are removed, resulting in a high-contrast “Warburg effect” image of aberrant tumor metabolism (Fig. [Fig Ch1.F4]e). The lipid suppression across all subjects was 90.5 ± 11.4 % with 66 % and 99 % of all skull pixels achieving suppression factors of at least tenfold and threefold, respectively. The signal retentions for Glc and Glx were 99.8 ± 7.5 and 100.2 ± 7.8 %, respectively. Lac retention cannot be quantitatively determined due to spectral overlap with lipid signals. However, the outlined simulation results (Figs. [Fig Ch1.F2] and [Fig Ch1.F3]) on high metabolite retentions together with a close visual agreement of Lac in Fig. [Fig Ch1.F4]c provide confidence that the Lac signal is preserved by the lipid removal algorithm.

## 4 Discussion

The use of spatial prior knowledge in DMI processing allowed for the removal of the extracranial lipid signals in datasets acquired of human head to below the spectral noise level. Similar approaches have previously been used for lipid signal removal in ^1^H MRSI (Dong and Hwang, 2006; Adany et al., 2016). However, ^1^H MRSI requires lipid signal suppression factors well in excess of 100. This puts much higher demands on accurate prior knowledge and signal homogeneity and may ultimately place a limit on the robustness for use on ^1^H MRSI data. In contrast, the low natural abundance of deuterium leads to much lower-amplitude lipid signals in the DMI data which were adequately removed with suppression factors below 10, thereby eliminating ambiguities in detecting elevated lactate levels. While lipid removal is desirable for all spatial positions to eliminate “hot spots” in the resulting lactate map (see Fig. [Fig Ch1.F4]d), it is especially prudent for skull areas immediately adjacent to the pathology (e.g., brain tumor) where partial volume effects would lead to a distorted lactate signal. The SLIM algorithm is based on spatial prior knowledge from high-resolution MRIs and is intrinsically suitable to separate skull from brain signals even in the presence of partial volume effects caused by the lower DMI resolution. This feature was systematically investigated with simulations (Fig. [Fig Ch1.F3]) and experimentally demonstrated (Fig. [Fig Ch1.F4]c) and confirmed constant lipid suppression performance in the absence or presence of partial volume effects.

The basic SLIM algorithm that uses two ROIs (one brain, one skull) without compensation for B_0_ and B_1_ magnetic field heterogeneity only provides modest lipid suppression (47 ± 21 % in simulations). Subdividing the skull ROI into smaller compartments quickly improves the lipid suppression to > 90, > 97, and > 99.5 % for ∼ 25, ∼ 35, and ∼ 45 ROIs, respectively. However, improved lipid suppression is accompanied by more variable metabolite retention as the SRF outside any given ROI becomes more dominant and localization increasingly relies on phase cancelation. The inclusion of B_0_ and B_1_ maps greatly improved the lipid suppression (to 92 ± 6 % in simulations for one brain and one skull ROI) without increased variability in metabolite retention. The use of surrogate B_0_ and B_1_ maps based on the ^2^H water shift and intensity provide a practical solution to the absence of high-resolution maps. Based on these results, it is recommended that B_0_ and B_1_ compensation should be used whenever possible, ideally in combination with a moderate subdivision of the skull ROI. It should be noted that while B_1_ heterogeneity was treated as a nuisance in the current implementation, it is possible to use B_1_ receive profiles for signal encoding to accelerate and/or improve data acquisition (An et al., 2011). While the average metabolite retention in the human brain in vivo is essentially 100 %, the standard deviations on Glc and Glx are somewhat higher than anticipated compared to simulation results (Fig. [Fig Ch1.F2]). Several factors can explain the increased variability. Firstly, removal of extracranial signals, including water and Glc, will perturb brain signals as voxel bleeding due to the MRSI point spread function is also removed. Secondly, MRSI voxels with significant partial volume effects will be perturbed as extracranial contributions are selectively removed. Thirdly, compensation of B_0_ and/or B_1_ heterogeneity with DMI-based surrogate B_0_ and/B_1_ maps will greatly decrease the variability in metabolite retention. However, since the surrogate maps are only an approximation of the actual B_0_ and B_1_ heterogeneity, the compensation is necessarily incomplete, thus leading to some residual variability. Finally, the signal-to-noise ratio (SNR) of in vivo DMI data is generally low, such that small perturbations in signal shape or noise level can lead to large relative changes in fitted signal amplitude.

In the current study the anatomical MRI and DMI datasets were acquired with the same RF coil assembly without subject movement. As a result, no spatial co-registration between MRI and DMI was necessary, and the MRI-derived spatial prior knowledge was accurate. However, in the presence of significant subject movement or when using MRIs acquired from a separate scan session (e.g., clinical MRIs), a mismatch between MRI-derived spatial prior knowledge and DMI can lead to greatly diminished lipid suppression performance and even a distorted metabolite profile. Simulations (data not shown) have indicated that a mismatch of 10 %–20 % of the nominal DMI voxel size can still retain a high level of lipid suppression, but that performance quickly degrades with larger mismatches. It is therefore recommended to acquire MRIs and DMI during the same session with an identical subject position. Spatial co-registration between MRI and DMI is possible but was not further pursued in this study. When subject movement during MRI/DMI scanning is anticipated, additional measures (e.g., motion tracking, additional immobilization) need to be taken to ensure accurate spatial prior knowledge.

The SLIM implementation as presented here for DMI can be modified and extended in several ways to enhance the immunity to signal heterogeneity and improve the reliability. Subdivision of the skull ROI can be optimized further. The number of ROIs and ROI shapes can be based on (1) optimizing the SRF contributions, (2) DMI-based lipid maps, or (3) high-resolution MRI-based lipid maps. The SLIM algorithm is also ideally suited to allow for regional analysis of brain ROIs during which ^2^H MR spectra can be obtained from anatomy-matched ROIs without partial volume effects. Development of automated definition of anatomical (GM, WM) and pathological (tumor core, tumor rim, FLAIR-enhanced) ROIs is currently in progress.

Similar levels of lipid suppression could have been achieved with alternative methods, such as dual-density reconstruction (Hu et al., 1994; Metzger et al., 1999), data extrapolation (Haupt et al., 1996) or L2 regularization (Bilgic et al., 2014). Whereas L2 regularization and data extrapolation can operate on standard MRSI data, dual-density reconstruction requires a modification of the data acquisition scheme, such that high *k*-space coordinates are sampled in addition to the standard low *k*-space coordinates typically acquired for MRSI. L2 regularization is based on the conditions of spatial and spectral orthogonality in which the desired and nuisance signals originate from spatially distinct compartments without spectral overlap. As the spectral overlap requirement cannot be fulfilled for lipids and lactate, L2 regularization will remove brain lactate signals, thus making it unsuitable for DMI. Like SLIM, data extrapolation only requires spatial separability of signals and would thus also be applicable to DMI. However, in the current investigation we focused on SLIM-based processing as the algorithm is readily extended to obtain signals from anatomy- and/or pathology-based ROIs.

## Supplement

10.5194/mr-5-21-2024-supplement The supplement related to this article is available online at: https://doi.org/10.5194/mr-5-21-2024-supplement.

## Data Availability

The MATLAB code to process 3D DMI data, referred to as DMIWizard, is freely available for download from GitHub via 10.5281/zenodo.10932507 (de Graaf, 2024). The experimental data are available through the Open Science Framework (OSF) repository at https://www.doi.org/10.17605/osf.io/64qzy (de Graaf et al., 2024).

## Appendix

The supplement related to this article is available online at: https://doi.org/10.5194/mr-5-21-2024-supplement.
