# Supplementary material for: Lipid removal in deuterium metabolic imaging (DMI) using spatial prior knowledge
Source: Magn Reson (Gott). 2024 Apr 9;5(1):21–31. doi: 10.5194/mr-5-21-2024 (PMC11836565; doi:10.5194/mr-5-21-2024)
Supplement: The supplement related to this article is available online at: https://doi.org/10.5194/mr-5-21-2024-supplement. [file mr-5-21-2024-supplement.pdf]

Supplement of Magn. Reson., 5, 21–31, 2024
https://doi.org/10.5194/mr-5-21-2024-supplement
© Author(s) 2024. CC BY 4.0 License.

Open Access
MAGNETIC RESONANCE

Supplement of

Lipid removal in deuterium metabolic imaging (DMI) using spatial prior knowledge

Robin A. de Graaf et al.

Correspondence to: Robin A. de Graaf (robin.degraaf@yale.edu)

The copyright of individual parts of the supplement might differ from the article licence.

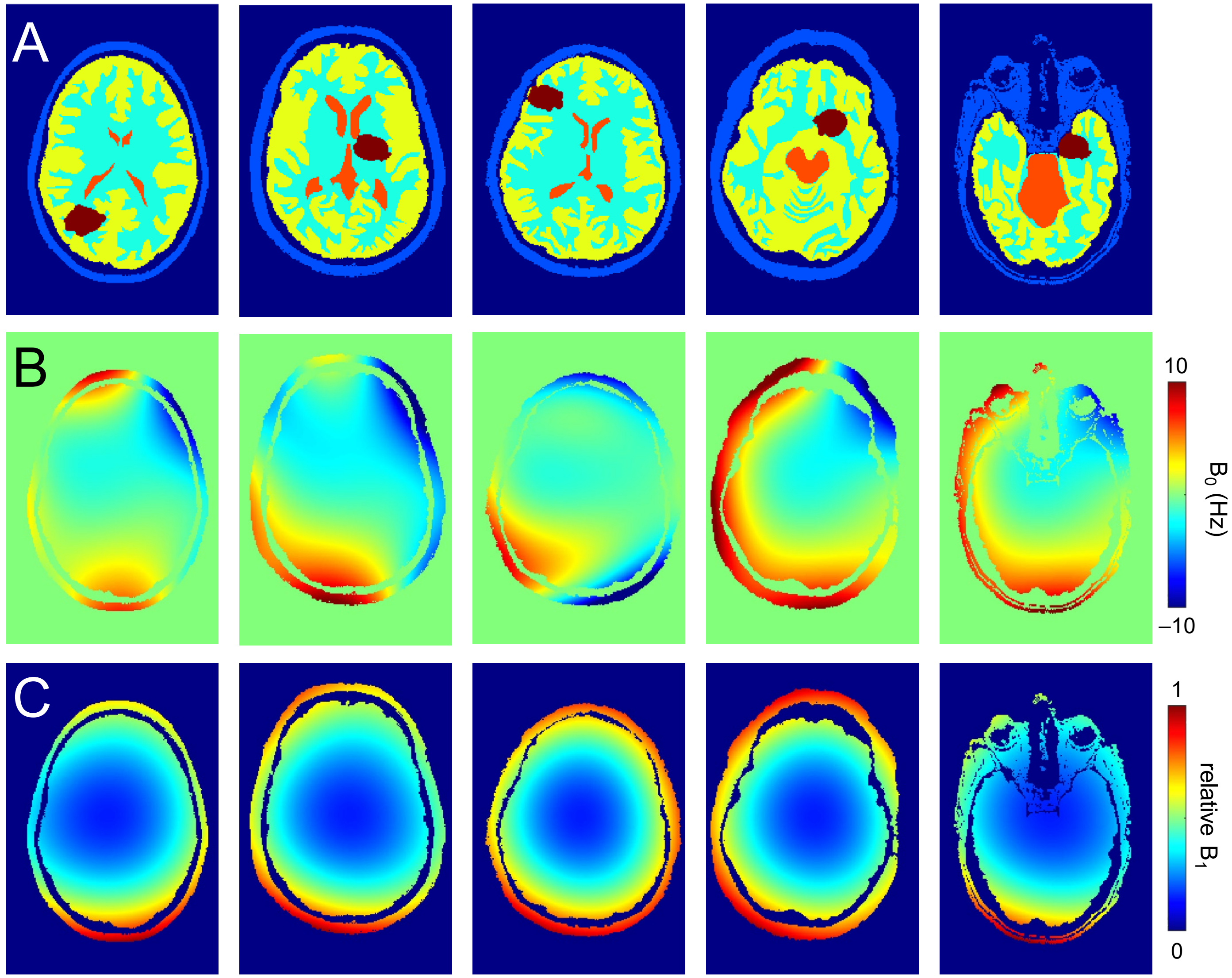

Figure S1 – Examples of brain and skull constellations used in simulations. (A) Brain and skull ROIs used in simulations on lipid suppression and metabolite retention. Brain ROIs of healthy volunteers were segmented into gray matter (GM), white matter (WM) and cerebrospinal fluid (CSF) after which a pathology ROI was placed at a random location. Each simulation was accompanied by randomly generated (B) B_0 and (C) relative B_1 maps based on ranges established *in vivo* (e.g., Fig. 1E/F). Anatomy-based ROIs were defined on five subjects, with ten permutations in pathology location and size and B_0/B_1 distribution per subject, for a total of 50 datasets.

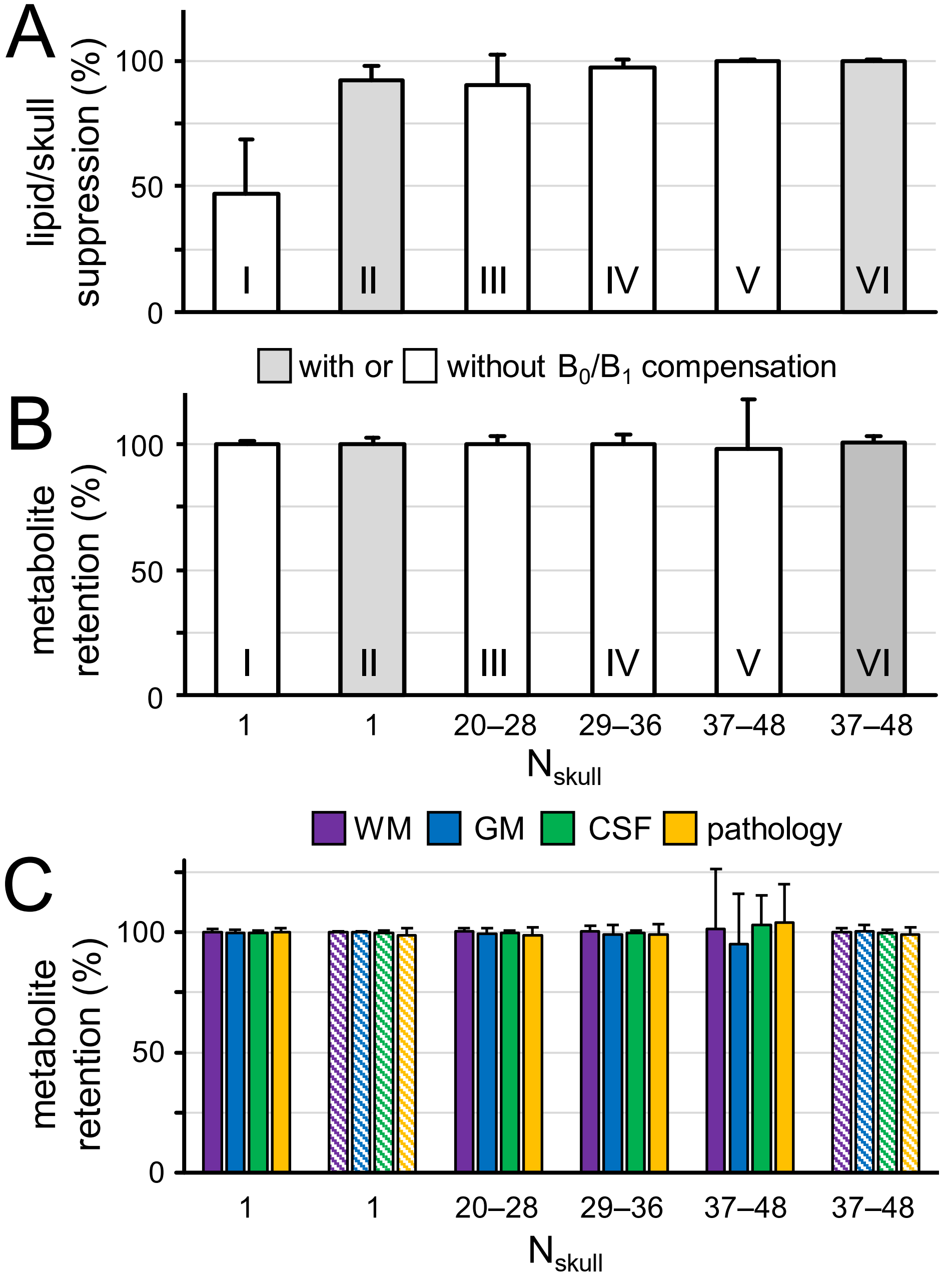

Figure S2 – Lipid suppression and metabolite retention for different skull ROI divisions. (A) Lipid suppression and (B) metabolite retention under different scenarios including N_{skull} = 1 without (column I) and with (column II) B_0 and B_1 compensation, N_{skull} = 20-28 (column III), 29-36 (column IV), 37-48 (columns V and VI) without (columns III-V) and with B_0 and B_1 compensation (column VI). Panels (A) and (B) are identical to Fig. 2G/H. (C) Metabolite retention for the separate brain ROIs (GM, WM, CSF, and pathology) under identical scenarios as in (A/B). Metabolite retention in separate brain ROIs (C) closely follow that across the entire brain (B).

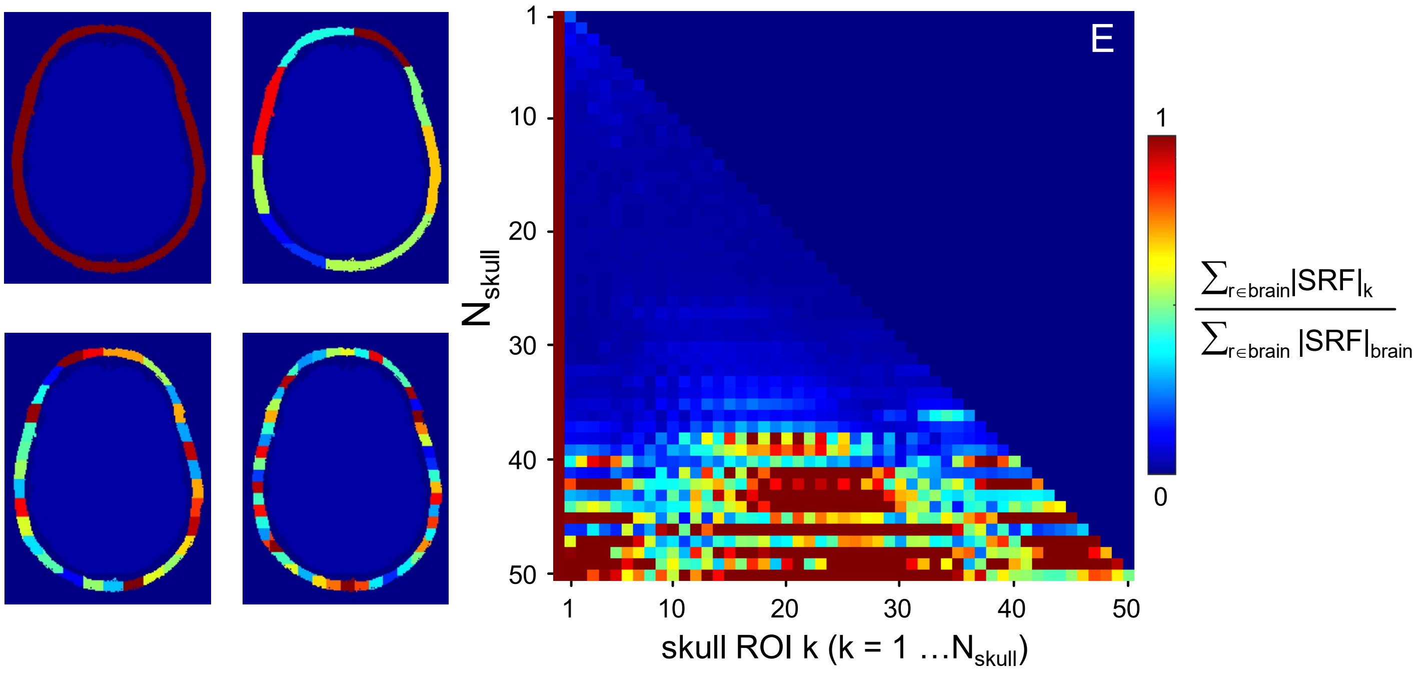

Figure S3 – Absolute-valued SRF contributions of varying number of skull ROIs to a single brain ROI. All calculations used the brain/skull constellation shown in Fig. 2 with the four brain compartments (GM, WM, CSF, pathology) combined into a single brain ROI. (A-D) Example skull subdivisions for N_{skull} = 1 (A), 10 (B), 30 (C) and 50 (D). The first (dark red) column represents the SRF contribution of the brain SRF to the brain ROI which is, by definition, 1.0 and is shown for intensity reference. For a coarse subdivision of the skull ROI (N_{skull} < 30-35), the SRF contribution of each skull ROI is low, in agreement with Fig. 2K. Note that summation over the *real-valued* SRF will produce a zero SRF contribution for all skull ROIs for any N_{skull}. The small *absolute-valued* SRF contribution seen for N_{skull} < 30-35 means that phase cancellation of signal across the brain compartment is only a minor contributor to the overall localization performance. While the skull SRFs are well-behaved, the small number of skull ROIs only provide modest immunity to B_0/B_1 heterogeneity (Fig. 2G, column III). Finer subdivision of the skull ROI (N_{skull} > 35) provides improved lipid suppression (Fig. 2G, columns IV and V), at the cost of greatly increased skull SRF contributions to the brain ROI (Fig. 2N). Even though phase cancellation provides perfect localization under homogeneous conditions, intra-compartment heterogeneity will lead to incomplete phase cancelation and to increased signal variation in the brain compartment (Fig. 2H, column V). The sharp transition from low to high SRF contributions at N_{skull} ~ 35 is similar for all 50 simulated brain/skull configurations.
